# A novel homozygous mutation in *GAD1* gene described in a schizophrenic patient impairs activity and dimerization of GAD67 enzyme

**DOI:** 10.1038/s41598-018-33924-8

**Published:** 2018-10-19

**Authors:** Chiara Magri, Edoardo Giacopuzzi, Luca La Via, Daniela Bonini, Viola Ravasio, Mohammed E. A. Elhussiny, Flavia Orizio, Fabrizio Gangemi, Paolo Valsecchi, Roberto Bresciani, Alessandro Barbon, Antonio Vita, Massimo Gennarelli

**Affiliations:** 1 Unit of Biology and Genetics, Department of Molecular and Translational Medicine, University of Brescia, Brescia, Italy; 2 Unit of Biotechnology, Department of Molecular and Translational Medicine, University of Brescia, Brescia, Italy; 3 Unit of Physics, Department of Molecular and Translational Medicine, University of Brescia, Brescia, Italy; 4 Neuroscience Section, Department of Clinical and Experimental Sciences, University of Brescia, Brescia, Italy; 5 Department of Mental Health, Spedali Civili Hospital, Brescia, Italy; 6 Genetic Unit, IRCCS Centro S. Giovanni di Dio Fatebenefratelli, Brescia, Italy

## Abstract

Recently, by whole exome sequencing of schizophrenia (SCZ) patients, we identified a subject that was homozygous for a novel missense substitution (c.391 A > G) in the glutamate acid decarboxylase 1 (*GAD1*) gene. *GAD1* encodes for GAD67 enzyme, catalyzing the production of gamma-aminobutyric acid (GABA) from L-glutamic acid. Here, we studied the impact of this mutation on GAD67 activity, dimerization and subcellular localization. Biochemical assay revealed that c.391 A > G reduces GAD67 enzymatic activity by ~30%, probably due to the impaired homodimerization of homozygous mutants as highlighted by proximity ligation assays. The mutational screening of 120 genes of the “GABAergic system” in a cohort of 4,225 SCZ cases and 5,834 controls (dbGaP: phs000473.v1.p2), did not identify other cases that were homozygous for ultra-rare variants in *GAD1*, but highlighted an increased frequency of cases that were homozygous for rare variants in genes of the GABA system (SCZ: 0.14% vs. Controls: 0.00%; p-value = 0.0055). In conclusion, this study demonstrates the functional impact of c.391 A > G variant and its biological effect makes it a good candidate as risk variant for SCZ. This study also supports an involvement of ultra-rare variants in GABAergic genes in the etiopathogenesis of SCZ.

## Introduction

Schizophrenia (SCZ) is a common psychiatric disorder with a strong genetic component^1^. Large case-control genomic studies have yielded substantial advances in clarifying the genetic architecture of the disorder. Results from genome wide association studies (GWASs) indicated that from one third to half of SCZ genetic contribution to variance in liability could be due to additive effects of a high number of common susceptibility alleles with modest effect size^2–9^. Whole exome sequencing (WES) and copy number variant (CNV) studies revealed that also rare variants play a role in schizophrenia^10–18^. These studies identified several candidate genes for SCZ, including genes involved in the glutamatergic (*GRM3*, *GRIN2A, GRIA1, ARC* and *NMDAR* complexes) and GABAergic neurotransmission (*GABAA* receptor complexes genes). These results suggest that an altered balance between excitatory glutamatergic and inhibitory GABAergic neuronal signaling could be one of the possible route to pathogenesis of SCZ.

Alongside studies that have dealt with the role of variants with an additive effect in SCZ, there are others that investigated the role of recessive variants. Even if a significant role of rare recessive deleterious variants did not emerge in the SCZ^19–21^, an enrichment of long Runs of Homozygosity (ROHs) has been found in SCZ cases^22,23^, suggesting that large autozygosity regions due to inbreeding could play a role in the disease. These last results are in line with our previous study^24^ pointing to the presence of rare SCZ risk variants, in a homozygous state, in subjects bearing long ROHs that are likely due to recent inbreeding.

Indeed, whole exome sequencing of SCZ patients with high levels of autozygosity, (that is with more than 22 Mb of their genome included in ROHs > 4 Mb, compared to a median value of 8.7 Mb)^24^, allowed us to identify some ultra-rare mutations in a homozygous state. These mutations, mapping in ROHs and affecting genes of the glutamatergic and GABAergic pathways, could be considered good candidate SCZ risk variants^24^. In particular, one of these variants, mapping at position chr2:171,687,546 (hg19 assembly) was a novel missense mutation (c.391 A > G) affecting the Glutamic Acid Decarboxylase I (*GAD1*) (NM_000817) gene. This gene encodes for the GAD67 enzyme, one of the two enzymes (the other is GAD65) that catalyze the production of gamma-aminobutyric acid (GABA) from L-glutamic acid. In human, GAD67 enzyme is mainly expressed in the brain, where it is constitutively active, contributing for ~90% of GABA basal levels^25^.

Reduced levels of *GAD1* mRNA and protein, as well as reduced GABA concentrations, have been consistently observed in multiple regions in *post-mortem* brains from SCZ cases^26,27^. In addition, histone modifications, changes in DNA methylation signatures, and altered spatial organization of the chromatin structure have been observed in *GAD1* promoter in the same tissues^28–31^. Some common polymorphisms in the proximal *GAD1* promoter have been found associated with increased genetic risk for SCZ, impaired working memory performance and accelerated loss of gray matter^32,33^. Finally, it has been shown that mice heterozygous for Gad67 mutations in GABAergic interneurons expressing Parvalbumin resemble several neurochemical and behavioral abnormalities observed in SCZ^34^.

The mutation previously identified in our schizophrenic patient results in the substitution of a threonine with alanine (p.Thr131Ala) in the first α-helix of the GAD67 N-terminal domain^35^; according to HOPE prediction software, the differences in dimension and hydrophobicity between the two amino acids will cause loss of hydrogen bonds in the protein core and disturb its correct folding^36^. We hypothesized that this mutation might result in the reduction of GABA production, a feature consistently observed in *post-mortem* brains from SCZ cases. To clarify the biological effect of this mutation, we studied its impact on GAD67 activity, dimerization and subcellular localization in *in vitro* cellular models and with molecular dynamics simulations. Moreover, to verify if homozygosity for rare variants in the *GAD1* gene and in genes of the GABAergic system could be considered a risk factor for SCZ, we measured the frequencies of homozygous subjects for rare variants in these genes in a cohort of 4,225 cases affected by SCZ and 5,834 controls from Sweden population (dbGaP: phs000473.v1.p2).

## Results

**Figure 1:**
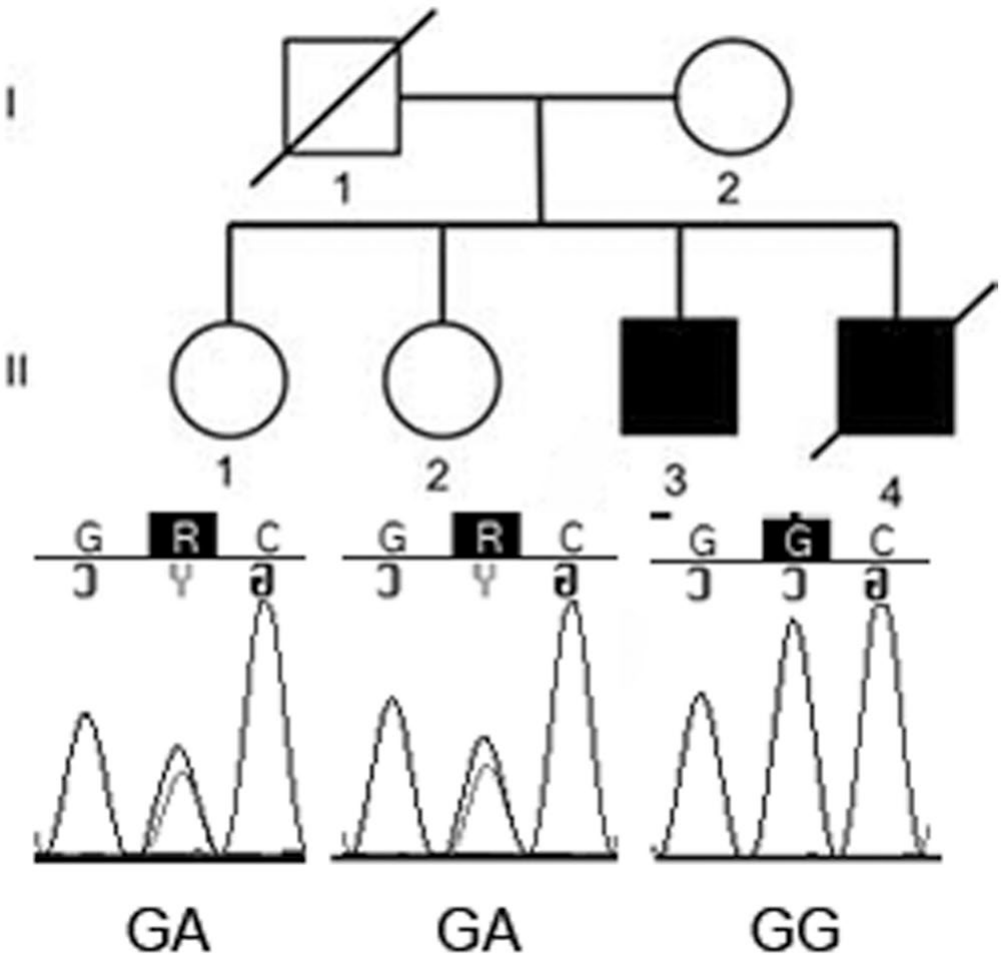
Pedigree and electropherograms of *GAD1* c.391 A > G mutation. Pedigree of the family: square indicates male and circle represent female; filled symbol means affected individual. The affected boy is homozygotes for the c. 391 A > G mutation, the two healthy sisters are heterozygotes.

Previously we identified a schizophrenic patient that was homozygous for a novel mutation in *GAD1* gene (*GAD1*, c.391 A > G). Since this mutation was predicted damaging by bioinformatics tools and fall in a gene whose expression has been described altered in some SCZ patients, we hypothesized it could be a risk variant for SCZ with a recessive effect^24^. Direct sequencing in the two healthy sisters of that patient revealed that both sisters were heterozygotes for the c.391 A > G mutation (Fig. 1). This result was in line with a recessive effect of the c.391 A > G mutation. Given the possible role of this mutation in the clinical phenotype of the patient, we decided to clarify its biological effect by functional studies.

### Functional Study of GAD1 c.391 A > G mutation

#### Intracellular localization seems not altered for GAD67 mutated protein

**Figure 2:**
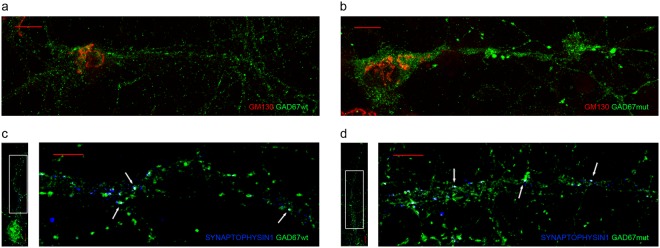
(**a**,**b**) Co-immunostaining of GAD67wt or GAD67mut (green) and Golgi Network (red) in transduced mice primary cortical neurons. (**c**,**d**) Single neuron and magnification of a dendritic region (white box) are reported. Co-immunostaining of GAD67wt or GAD67mut (green) and Synaptophysin1 (blue). Scale bar = 10 μm.

To test if the c.391 A > G mutation could alter intracellular trafficking of GAD67, we performed immunofluorescence experiments on mice cortical neurons transduced with lentiviral vectors expressing GAD67 wild type (GAD67wt) or GAD67 with the p.Thr131Ala mutation (GAD67mut). GAD67mut showed a cellular localization similar to GAD67wt. As shown in Fig. 2(a,b) and Supplementary Fig. 1, they showed a vesicular clusterization in cytoplasm and neurites. Co-staining with synaptophysin highlighted that both isoforms were localized at presynaptic vesicles (Fig. 2c,d).

**Figure 3:**
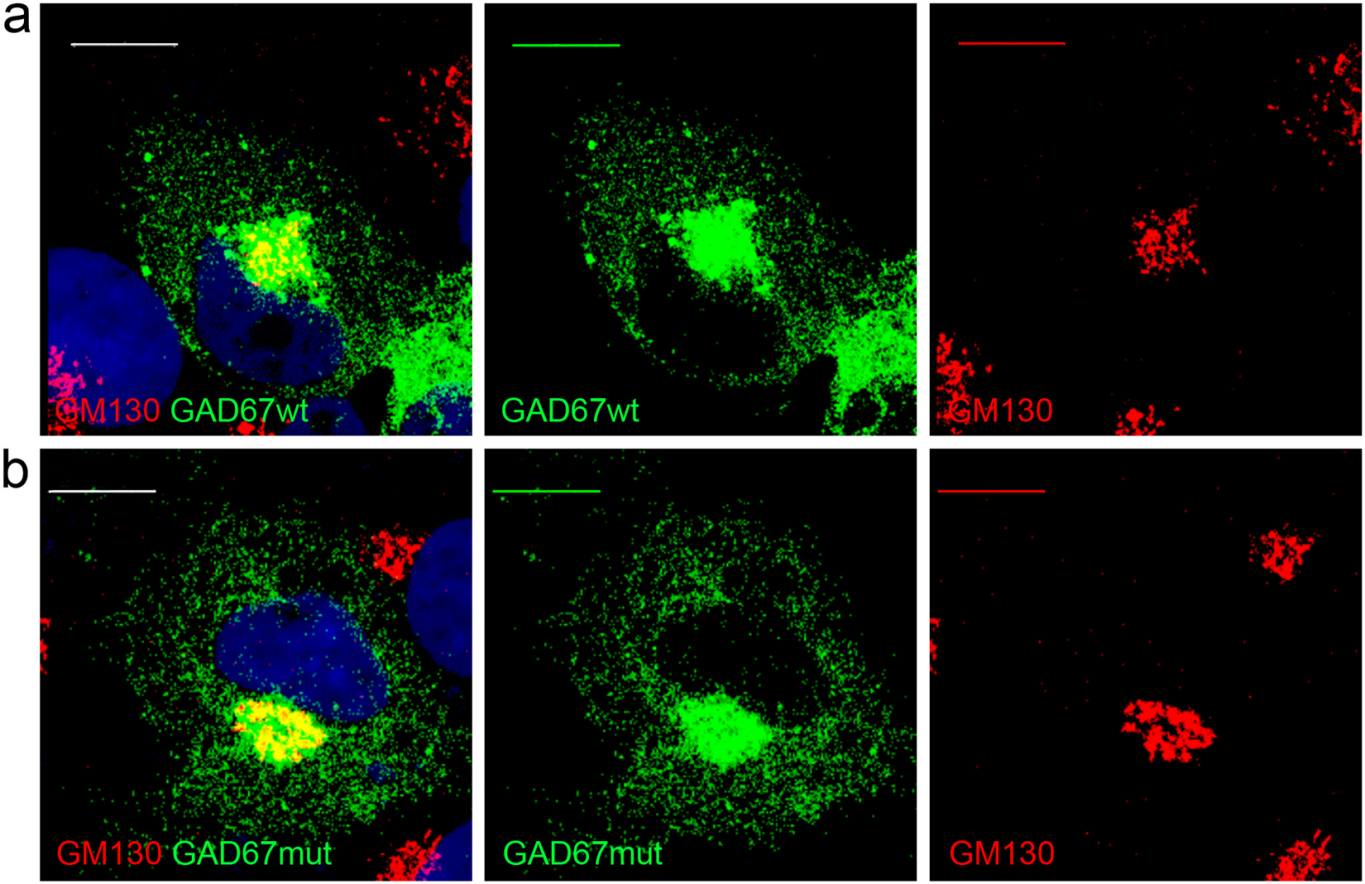
Co-immunostaining of GAD67wt (**a**) or GAD67mut (**b**) (green) and Golgi Network (red) in transfected COS7 cells. Nuclei were stained using Dapi (blue). Scale bar = 10 μm.

Recently a mutation in GAD67 N-terminal domain was shown to abolish GAD65-independent membrane anchoring of GAD67 in primary neurons, without affecting GAD65- dependent membrane anchoring mechanism^37^. To investigate if the p.Thr131Ala mutation could abolish GAD65-independent membrane anchoring mechanism, we repeated localization experiments in COS7 cells. This cell line does not express endogenous GAD65 and has been previously demonstrated as a model to study the GAD65-independent membrane anchoring mechanism^37^. Even in this cell line, GAD67wt and GAD67mut showed a proper cellular localization. GAD67wt and GAD67mut were targeted to Golgi membranes and to cytoplasmic vesicles (Fig. 3 and Supplementary Fig. 2).

#### GAD67 mutated protein showed reduced enzymatic activity

**Figure 4:**
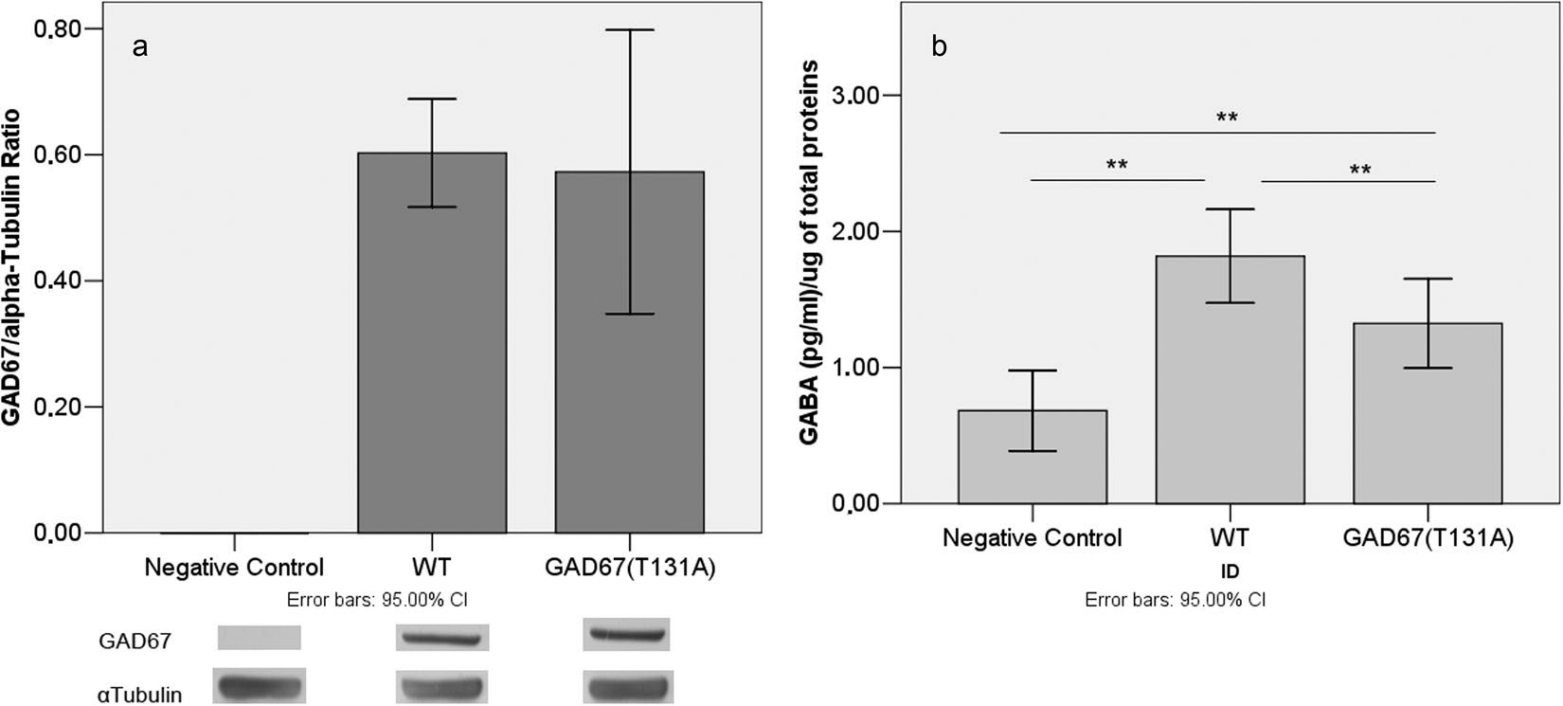
Average amount of GAD67 protein and GABA production. (**a**) Densitometric analysis of Western Blot. Histograms report average amount of GAD67 normalized to alpha-Tubulin protein. Each histogram bar represents the mean value of triplicates. Cropped images of WB bands are reported below the graph, the original image of WB stain is given in Supplementary Fig. 5. (**b**) Histograms depict average amount of GABA normalized to total proteins amount measured. Each histogram bar represents the mean value of triplicates. Significance estimated by one-way ANOVA with LSD post hoc test (**P < 0.01).

To investigate the effects of p.Thr131Ala substitution on GAD67 enzymatic activity, we measured the amount of GABA produced by GAD67wt and GAD67mut using an *in vitro* biochemical assay. As shown by Western Blot (WB), GAD67wt and GAD67mut proteins were present at comparable amount in lysate samples from transfected HEK293T cells. As shown in Fig. 4, the amount of GABA produced by transfected cells was higher than in negative control. The amount produced by the GAD67 mutated enzyme, however, was 33% less than that produced by the wild type isoform (GAD67mut/GADwt ratio = 0.67; Least Significant Difference (LSD) p-value = 0.0035).

This result was than confirmed in an independent experiment (GAD67mut/GADwt ratio = 0.73 LSD p-value = 0.0333).

#### GAD67 mutant shows impaired homodimerization, but it is able to form heterodimers with GAD67 wt protein

GAD67 enzyme requires dimerization for proper activation^38–41^. To investigate if p.Thr131Ala mutation could impair homodimerization of GAD67, we performed PLA assays in HEK293T co-transfected with various combinations of GAD67wt and GAD67mut constructs. In particular, we co-transfected HEK293T cells with: GAD67wt monomers tagged with either a HA or MYC epitope (GAD67wt/wt); GAD67wt and GAD67mut monomers tagged with HA and MYC (GAD67wt/mut), respectively; GAD67mut monomers tagged either with a HA or a MYC epitope (GAD67mut/mut).

**Figure 5:**
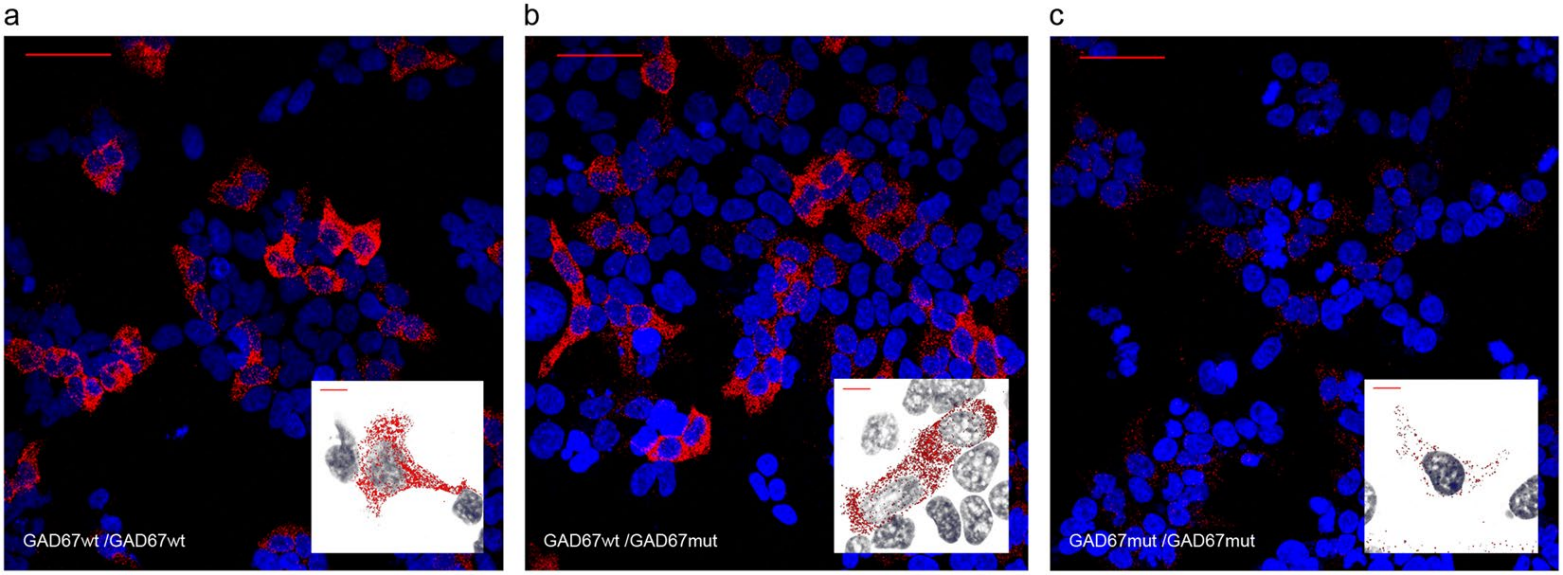
Proximity Ligation Assay of GAD67 homodimerization in GAD67wt/wt, GAD67wt/mut, GAD67mut/mut HEK293T transfected cells. (**a**) Red dots represent dimerization events between GAD67wt monomers; (**b**) red dots represent dimerization events between GAD67wt and GAD67mut monomers; (**c**) red dots represent dimerization events between GAD67mut monomers. Scale bar = 40 μm: In the white boxes magnification of representative cells are reported. Scale bar = 10 μm.

A higher number of PLA positive signals was observed in HEK293T cell lines co-transfected with GAD67wt/wt and GAD67wt/mut compared to GAD67mut/mut. (Fig. 5). As reported in Table 1, GAD67mut/mut cells had a significantly reduced number of homodimerization signals compared to GAD67wt/wt and GAD67wt/mut cells, whereas the number of homodimerization signals measured in the GAD67wt/wt and GAD67wt/mut cells were not statistically different. This result was confirmed in an independent experiment.

**Table 1:** Comparison of PLA positive signals among HEK293T cells co-transfected with various combinations of GAD67wt and GAD67mut constructs.

Mean PLA spots/cell (SD)			ANOVA	Comparison Group	LSD p value
GAD67_wt/wt_	GAD67_wt/mut_	GAD67_mut/mut_			
82 (11.3)	78.7 (10.5)	34 (5.7)	*F*_*2,6*_ = 24.094 *p* = 0.0014	GAD67_wt/wt_ vs GAD67_wt/mut_	0.681
				GAD67_wt/wt_ vs GAD67_mut/mut_	0.001
				GAD67_wt/mut_ vs GAD67_mut/mut_	0.001

#### Molecular dynamics simulations

The effect of the p.Thr131Ala mutation on the structure of GAD67 was also investigated by the use of molecular dynamics (MD). The mutation causes a local modification of the secondary structure: a β-sheet component is observed in the mutant around residues 140 and 180, probably related to the loss of a hydrogen bond between Arg 181 and residue 131 (Supplementary Fig. 3). A change in the shape of the N-terminal region, up to residue 210, was also found in the MD simulations (Supplementary Fig. 4). These observations are compatible with a structural modification that could affect the dimerization mechanism.

### *In silico* screening of rare variants in a homozygous state in genes of the GABA system

#### Increased frequency of homozygous cases for rare variants in genes of the GABA system

To investigate the role of ultra-rare variants in a homozygous state in *GAD1* gene and in genes involved in the GABAergic system as risk factors for SCZ, we measured their frequency in a database of 4,225 schizophrenic cases and of 5,834 controls from Sweden population (dbGaP: phs000473.v1.p2). We did not identify any other subject being homozygous for ultra-rare variants in *GAD1*. However, when the analysis was extended to 119 autosomal genes of the GABA system, we observed a significant higher frequency of cases that were homozygous for rare mutations (Minor Allele Frequency (MAF) in the dataset < 0.001) compared to controls (One-tailed Fisher exact test p-value = 0.0055). The difference remained significant even when only mutations with a frequency lower than 0.01 or 0.001 in known human populations from ExAC and 1000 G were retained for the analyses (p = 0.013 and p = 0.0311, respectively). No significant association (p = 0.0741) was observed, instead, when only likely disruptive ultra-rare mutations were considered. In this latter case, however, the considered dataset was largely under-powered (power = 33%) to detect such a small effect. Detailed results are reported in Table 2 and the identified mutations in Table 3.

**Table 2:** Number (N) of cases and controls that are homozygous for rare mutations in genes of the “GABA system”. P-values of the one-tailed Fisher exact tests and of the simulation tests are also reported.

Mutation Category	N. of SCZ cases (%) (Total = 4,225)	N. of Controls (%) (Total = 5,834)	One-tailed Fisher’s test p value	Simulation p value
Dataset MAF 0.001 No population filter	6 (0.14%)	0 (0.00%)	0.0055	0.0408
Dataset MAF 0.001 Population MAF 0.01	5 (0.12%)	0 (0.00%)	0.0130	0.0428
Dataset MAF 0.001 Population MAF 0.001	4 (0.09%)	0 (0.00%)	0.0311	0.0482
Dataset MAF 0.001 Population MAF 0.001 Likely disruptive	3 (0.07%)	0 (0.00%)	0.0741	—

“Dataset MAF” refers to minor allele frequency in the Sweden dataset after filtering, while “Population MAF” refers to the maximum values reported in human populations from ExAC, ESP and 1000 G. Likely disruptive mutations are defined as missense variants with CADD > 20, nonsense substitutions and splice junction mutations. Simulation p values were calculated as the fraction of the 5,000 simulations obtaining a difference in the frequency of homozygotes equal to or greater than that observed in the original dataset (see methods).

**Table 3:** List of rare mutations in genes of the “GABA system” that has been found in a homozygous state among cases or controls.

Genomic	A/U	Gene	Mutation class	cDNA	Aa change	Dataset MAF (N. individuals)	Population MAF	CADD
chr2:25044490 T > G	1/0	*ADCY3*	missense	NM_004036:c.3555 A > C	E1008A	7.46E-04 (10,055)	2.55E-04	20.5
chr5:170236670 G > A	1/0	*GABRP*	missense	NM_014211:c.1128 G > A	G311R	2.98E-04 (10,058)	9.01E-05	32
chr8:131880120 C > G	1/0	*ADCY8*	missense	NM_001115:c.2182 G > C	A728P	9.94E-05 (10,056)	1.00E-03	18.29
chr11:122929541 T > C	1/0	*HSPA8*	Splice junction	NM_153201:c.1601-3 A > G		9.94E-04 (10,055)	3.33E-04	0.66
chr16:76532538 G > A	1/0	*CNTNAP4*	missense	NM_033401:c.2311 G > A	R770Q	2.50E-04 (9,986)	7.52E-05	21.7
Chr17:39881003 C > T	1/0	*HAP1*	missense	NM_177977:c.1810G > A	G604R	1.49E-04 (10,059)	5.22E-02	0.03

“Dataset MAF” refers to minor allele frequency in the Sweden dataset after filtering, while “Population MAF” refers to the maximum values reported in human populations from ExAC, ESP and 1000 G. A/U: Number of Affected/Unaffected subjects with mutations in a homozygous state. Splice junction refers to variants located in the 3 bp at exon/intron boundaries.

To verify if a difference equal to or greater than that observed between cases and controls could be obtained by chance analyzing any group of 120 genes of the genome, we performed a simulation. The results of simulation revealed that cases tended to be more homozygous than controls for rare variants in genes of the GABA systems compared to other genes of the genome (simulation p-values reported in Table 2). This data suggested that the higher frequency of mutations in a homozygous state observed among cases was specific of the genes of the “GABA system” and was not due to a generalized enrichment of ultra-rare variants in a homozygous state in the genome of schizophrenic cases. This was further confirmed by assessing the frequency of cases and controls that were homozygous for at least one ultra-rare mutation in any gene of the genome excluding the 120 genes of the GABAergic system. No significant difference in the frequency of homozygous subjects between cases and controls was observed (One-tailed Fisher’s p = 0.078).

## Discussion

In a recent study aimed to shed light on the role of autozygosity and recessive variants in SCZ, we identified a patient that was homozygous for a novel missense mutation (c.391 A > G) mapping at position chr2:171,687,546 (hg19 assembly) in the *GAD1* gene (NM_000817) and predicted to be damaging by bioinformatics tools^24^. Here, through a detailed functional characterization, we demonstrated the functional effect of the c. 391 A > G variant. Moreover, by in silico analysis, we brought new findings suggesting the role of rare variants in a homozygous state in the GABAergic system as risk factors for SCZ.

Intracellular localization experiments revealed that the c.391 A > G mutation does not impair subcellular localization of GAD67 that is properly detected at the level of presynaptic vesicles. Although the mutation is in the N-terminal domain, it does not impair the GAD65-indipendent membrane anchoring mechanism mediated by this domain^37^. Indeed, GAD67mut isoform shows a proper subcellular localization also in cell types not expressing GAD65 enzyme.

Biochemical assays revealed that the amino acid substitution p.Thr131Ala (induced by c.391 A > G) reduces GAD67 enzymatic activity by ~30%. This effect is not due to increased degradation of the mutated protein, but to changes in dimerization properties, as suggested by our results from PLA experiment and molecular dynamics simulations. Indeed, the native structure of GAD67 is a dimer connected by noncovalent linkages^39–41^ and homodimerization or heterodimerization with GAD65 are critical for enzymatic activity since the active site of GAD is formed by the interaction of the two GAD monomers^39^. Intriguingly PLA results revealed that the p.Thr131Ala mutation impairs homodimerization only when present in a homozygous state, demonstrating that the *GAD1* c.391 A > G mutation exerts a recessive effect. This result is in line with the recessive effect of the mutation suggested by pedigree analysis. Indeed, the affected proband is homozygous for the c.391 A > G mutation, whereas the two healthy sisters are heterozygous.

GABA is the main inhibitory neurotransmitter in mammals, it is secreted by inhibitory interneurons and it is responsible for regulating the excitability of other neurons, including glutamatergic ones. Several studies found low *post-mortem* concentrations of this neurotransmitter in several brain regions of schizophrenic patients^42–47^. The observed low levels of GABA in the brain seem to be related to altered expression of *GAD1* gene, given that reduced expression of *GAD1* mRNA and GAD67 protein have been reported in multiple brain regions of schizophrenic patients (a review of these studies could be found in^26,27^).

Engineered mice models further corroborate the hypothesis that decreased expression of *GAD1* gene is implicated in SCZ. Mice completely lacking *GAD1* gene died of severe cleft palate shortly after birth, whereas hemizygous mice for *GAD1* deletion survived and showed about 35% reduction of GABA concentration in the cerebral cortex^25^. Moreover, in conditional GAD67 KO-mice, in which GAD67 was deleted only in a subset of GABAergic neurons, haploinsufficiency of GAD67 induced SCZ-related phenotype and synaptic dysfunction^34^. In particular, conditional GAD67 KO-mice were more sensitivity to the locomotor-stimulating effects of MK-801, they showed impaired prepulse inhibition and deficits in social memory compared to WT mice. Moreover, they showed a decreased number of PV neurons in the cerebral cortex, altered properties of NMDA receptor-mediated synaptic responses in pyramidal neurons and an increased spine density in hippocampal CA1 apical dendrites.

Taken together, these data suggests that dysregulated *GAD1* expression in specific brain areas may concur to SCZ phenotype. In this perspective, the fact that the *GAD1* c.391 A > G mutation identified in our patient induces a ~30% reduction of GABA synthesis makes it a good candidate as SCZ risk variant.

Interestingly, the c.391 A > G mutation not only affects GAD67 primary sequence, but also primary sequence of GAD25, another *GAD1* transcript derived from alternative splicing^48^. GAD25 is predominantly expressed during embryonic stages^48,49^ and it has been proposed to play a role in developmental processes, such as cell proliferation, migration, and/or synaptogenesis^50^. *GAD1* expression in human prefrontal cortex increases during development, from late prenatal period to early adolescence and adulthood^30^, along with a progressive switch from GAD25 to GAD67^51^. Thus, the possibility that the c.391 A > G mutation might impair not only the release of GABA in adult brain, but also influence brain developmental processes is an intriguing hypothesis. Indeed, according to the neurodevelopmental hypothesis of SCZ, the etiology of the disorder may involve pathologic processes, caused by both genetic and environmental factors, beginning early in life and leading to an abnormal activation of neural circuits during adolescence or young adulthood^52^.

We are aware that effects of c.391 A > G mutation have been demonstrated only in *in vitro* models and compensatory mechanisms could act in the GABAergic interneurons, where GABA is produced not only by GAD67, but also by GAD65^49^. The two enzymes, however, seem to have distinct functions with GAD67 as the only responsible for basal GABA production^38^. In contrast, GAD65 is transiently activated in response to the demand for extra GABA in neurotransmission and cycles between an active holo form and an inactive apo form^38^. For these reasons, evaluation of actual impact of this mutation in a complex system, like brain is, would be an interesting perspective for further investigation.

As secondary aim of the study, we wanted to clarify the role of ultra-rare variants in a homozygous state in *GAD1* gene and in genes involved in the GABAergic system as risk factors for SCZ. Thus, we measured their frequency in a large cohort of 4,225 SCZ cases and 5,834 controls (dbGaP: phs000473.v1.p2 database). The results suggest an involvement of these mutations as risk factor for SCZ. Indeed, although we did not identify any other homozygous subjects for rare variants in the *GAD1* gene, we observed, among SCZ cases, an increased frequency of homozygous subjects for rare mutations in genes related to the GABAergic system compared to controls. Due to the low number of rare variants identified in a homozygous state, however, a larger sample size would be required to obtain a more robust statistic. Moreover, the mutations reported in the analyzed database were not confirmed by alternative methods (such as Sanger sequencing). Therefore, despite the stringent quality controls applied to variant dataset and the results of simulation analysis, we could not completely exclude that the observed burden is inflated by genotyping errors (i.e. heterozygotes being incorrectly called as homozygotes).

Deficits in GABAergic signaling have long been hypothesized to contribute to SCZ pathophysiology, but have often been considered adaptive responses resulting from a broad range of genetic, biological and environmental factors^26,53,54^. Our results suggest that alterations in GABAergic signaling may have direct causal relevance for SCZ, rather than be a secondary effect. This hypothesis is in agreement with the study of Pocklington^17^, demonstrating that CNVs found in schizophrenic cases are enriched for genes involved in GABAergic neurotransmission. It is also in line with the study of Balan^55^, suggesting SNPs in the GABAergic system as SCZ risk factors.

In conclusion, this study characterizes for the first time the functional effect of a mutation in *GAD1* gene detected in a schizophrenic patient and provides clues suggesting rare variants in a homozygous state in the GABAergic system as driver events in the etiopathogenesis of SCZ. Further studies performing targeted re-sequencing of GABA related genes in larger cohorts will provide better evidences on the role of ultra-rare mutations in this pathway in SCZ pathogenesis.

## Materials and Methods

### Samples description and mutation screening

The homozygous patient for the c.391 A > G mutation is a 42-year-old male satisfying the DSM-IV-TR criteria for SCZ with no psychiatric disorders in comorbidity. He belongs to the SCZ cohort analyzed in the following papers:^12,56^; his clinical and demographic characteristics are described in the supplemental materials of^24^. This patient belongs to a group of seven unrelated SCZ patients that were carriers of a high number of long ROHs indicating that they were children of consanguineous parents. In particular, the patient under investigation has 49 ROHs larger than 1 Mb in his genome, that contain 1,711 variants in a homozygous state. The c.391 A > G mutation in *GAD1* was the only novel missense variant falling in a conserved region and predicted damaging.

The family of the patient was composed of four siblings: the proband, another brother with SCZ (dead at the time of this study) and two healthy sisters (Fig. 1). The exclusion of SCZ in the two sisters was assessed with a clinical structured interview for disorders of Axis I DSM-IV (SCID-I).

The two healthy sisters were screened for the presence of the c.391 A > G mutation using Sanger sequencing and specific primers *GAD1*-F and *GAD1*-R (Supplementary Table 1). Informed consent for the study was obtained from all participants.

All genetic analyses were approved by the local Ethic Commitee (NP1581-01/14/2014) and all experiments were carried out in accordance with relevant guidelines and regulations.

### Generation of transfection/transduction vectors for cellular assays

Six different constructs containing wild-type or mutated isoforms of *GAD1* coding sequence were used for transfection/transduction in cellular and enzymatic assays. Main details of each construct are reported in Supplementary Table 2.

### GAD67 expression vectors for enzymatic activity assay

Starting from 50 ng of human brain cDNA, the *GAD1* coding sequence (NM_000817) was amplified using Takara PrimeSTAR (Takara) with specific primers (Supplementary Tables 1 and 2) and cloned in the pIRES-GAD67-hrGFP construct expressing GAD67 and GFP as independent proteins (pGAD67-wt) (Supplementary Table 2).

Starting from 50 ng of pGAD67-wt constructs, the QuickChange II site-directed mutagenesis kit (Agilent) was used to introduce the desired mutation c.391 A > G. The template DNA was amplified using specific mutagenesis primers (GAD67_A391G_F and GAD67_A391G_R, see Supplemental Table 1) following the manufacturer’s indications and the amplification mix was used to transform *E. coli* XL-1 Blue competent cells. The final mutated construct (pGAD67-mut) was verified by Sanger sequencing. Both pGAD67-wt and pGAD67-mut constructs were used for biochemical assay.

#### GAD67 expression vectors for sub-cellular localization study and PLA assay

Starting from the pGAD67-wt and pGAD67-mut construct described above, *GAD1* wild type and *GAD1* c.391 A > G isoforms were sub-cloned in pRRLSIN.cPPT.PGK-EGFP.WPRE (ADDGENE #12259). The EGFP coding sequence was removed from the vector and substituted with *GAD1* coding sequence. *GAD1* wt and mutated coding sequences were amplified with specific primers to add HA or Myc tags (Supplemental Table 1) at protein C-terminus. Four different constructs were obtained: pRRLSIN-GAD67wt-HA; pRRLSIN-GAD67mut-HA; pRRLSIN-GAD67wt-Myc; pRRLSIN-GAD67mut-Myc (Supplementary Table 2).

#### Virus generation for transduction in sub-cellular localization study and PLA assay

Virus particles were generated by calcium phosphate transfection of HEK293T cells in 100-mm Petri dishes. 24 h before transfection, cells were plated at a density of 30,000 cell/cm^2^ in 24-well plates; medium was changed 2 h before transfection. 0.5 µg of the plasmid of interest were mixed with 0.1×Tris/EDTA (TE 0.1×)/dH2O (2:1) and 2.5 M CaCl_2_; mixture was maintained 5 min at RT. Precipitate was formed by adding dropwise 2× HBS solution to the mixture, then suspension was added immediately to cells. Calcium-phosphate plasmid DNA mixture was allowed to stay on cells for 14–16 h and then replaced with fresh medium.

Independent reactions were prepared using 32 μg of either pRRLSIN-GAD67wt-HA; pRRLSIN-GAD67mut-HA; pRRLSIN-GAD67wt-Myc; pRRLSIN-GAD67mut-Myc, plus 7 μg pMD2.G (ADDGENE #12259), 16.25 μg pCMVR8.74 (ADDGENE #22036), and 6.25 μg pRSV-Rev (ADDGENE #12253). Two supernatants were collected 24 and 48 h post transfection and centrifuged at 42,425 × *g* 2 h at 4 °C. Pellet was resuspended in DPBS (Gibco), aliquoted and stored at −80 °C. Viral titer was checked in serial dilutions on HEK293T cells. MOI 5 was used to transduce mouse cortical neurons at DIV18, and HEK293T cells in subsequent experiments.

### Study of subcellular localization for GAD67 wt and mutated proteins

For analyses of the subcellular localization of GAD67 wt and mutated isoforms, immunofluorescence experiments have been performed both on transduced primary cortical neuronal cells and transfected COS7 cells. Cells were transduced with lentiviral particles derived from pRRLSIN-GAD67wt-HA; pRRLSIN-GAD67mut-HA constructs, in independent experiments. The same expression vectors were used for transfection experiments (Supplementary Figs 1 and 2). Details about preparation and transfection of mouse primary cortical cultures and COS7 cell lines cultures are reported in Supplementary Materials.

### Immunofluorescence staining and image analysis

After transduction or transfection, cells were fixed in 4% PFA in phosphate-buffered saline (4% PFA–PBS, Life Technologies, Invitrogen) for 15 min at room temperature and permeabilized in PBS containing 0.3% Triton X-100 (Sigma-Aldrich) for 10 min. Subsequently, cells were rinsed in PBS and washed twice with 0.15 M glycine in PBS for 5 min. Cells were pre-incubated in blocking solution (Roche Applied Science) at room temperature (RT) for 45 min and then further incubated with primary antibodies diluted in blocking solution at RT for 1 h. The primary antibodies used were rabbit anti-HA (SIGMA; cod. H6908) diluted 1:100, to recognize GAD67wt and GAD67mut, mouse anti-GM130 (Becton Dickinson, BD) diluted 1:300, to recognize Golgi Complex/Apparatus and mouse anti-synaptophysin (Synaptic System) diluted 1:300, conjugated with Alexa-Fluor 647, to recognize presynaptic sites. Secondary antibodies conjugated with Alexa-Fluor 488 or Alexa-Fluor 594 dyes (Life Technologies, Invitrogen) were used diluted 1:500. Nuclei were detected using DAPI staining. Images were acquired on Zeiss LSM 510 Meta confocal microscope (Carl Zeiss, Milan, Italy).

### Enzymatic activity assay on GAD67 wt and mutated isoforms

To assess the enzymatic activity of GAD67 wt/mutated isoforms, we used a specific GABA ELISA assay (Cloud-Clone Corp.) to measure the amount of GABA produced by cells extracts derived from pGAD67-wt and pGAD67-mut transfected HEK-293 cells.

### Cell transfection

For transient transfection, cells were seeded in 60 mm diameter Petri dish and incubated for 24 h in serum-free medium (OptiMEM, Gibco-BRL) with 2 μg of plasmid DNA and FuGENE HD reagent (Promega) in ratio 1:3. After 24 h, serum-free medium was removed and cells were incubated in DMEM 10% FBS for another 24 h. Transfected cells were then washed in PBS (Gibco-BRL), collected by scraping, resuspended in PBS (Gibco-BRL) + PLP 0,2 mM, and subjected to mild sonication (two 10″ pulse at 4 °C, 10% power, using a probe Sonicator Sonoplus-Bandelin electronic). Supernatant obtained after a centrifugation at 800 g for 10 min represented the cell extract and was used for protein quantitation, enzyme assay and Western-blot analysis.

### GAD67 *in vitro* enzymatic assay

Pre-warmed (37 °C) assay buffer (50 mM KPO4 pH 8.0, 0.2 mM PLP, 1 mM AET, 100 μg/mL BSA) containing 20 mM sodium glutamate was added to test tube containing the different cell lysates in ratio 1:1. After 120 minutes of incubation at 37 °C, samples were immediately placed in ice to stop the reaction and GABA was immediately quantified. Each determination was performed in triplicates and the whole experiments were repeated twice.

#### Statistical methods for the analysis of enzymatic assay data

Concentration of GABA was measured as pg/ml and normalized on total amount of proteins in cell lysate. Comparison of GABA levels among not transfected cells (negative control), pGAD67-wt and pGAD67-mut transfected HEK-293 cells was performed using ANOVA test followed by multiple comparisons t-tests (Fisher’s Least Significant Difference (LSD) tests).

### Western Blotting (WB)

To determine amount of GAD67 enzyme, cell extracts were subjected to 12% (w/v) SDS-PAGE and subsequently transferred by electroblotting onto Hybond-P blotting membrane (GE Life Sciences). Membranes were blocked for 1 h in PBS containing 5% (w/v) dried milk and subsequently incubated for 1 h RT with goat anti-*GAD1* primary antibody (Everest Biotech EB09109) diluted 1:500 in PBS + 0,1% (v/v) Tween 20 + 1% (w/v) dried milk. After several washes with PBST, membranes were incubated for 45 min (RT) with donkey anti-goat HPR-conjugated secondary antibody (Santa Cruz), diluted 1:5000 in PBST. Detection of immunocomplexes was carried out using SuperSignal West Pico Chemiluminescent Substrate detection kit (Thermo Scientific). Intensity of immunoreactive bands was analyzed with Image-Pro Plus. Data are presented as optical density ratios of investigated protein band normalized for alpha tubulin bands in the same line.

### Proximity Ligation Assay (PLA)

The Duo-link® PLA Technology® kit (Sigma-Aldrich) was used to assess homodimerization of GAD67 complexes. HEK293T cells were transduced, in different experiments, with the viral particle derived from constructs: a) pRRLSIN-GAD67wt-HA and pRRLSIN-GAD67wt-Myc; b) pRRLSIN-GAD67wt-HA and pRRLSIN-GAD67mut-Myc; c) pRRLSIN-GAD67mut-HA and pRRLSIN-GAD67mut-Myc.

24 h before transduction, cells were plated at a density of 30,000 cell/cm^2^ in 24-well plates; medium was changed 2 h before the experiments. MOI 5 was used to transduce HEK293T, adding viral particles directly to the culture media.

PLA was performed accordingly to the manufacturer’s instructions with minor modifications (Supplementary Materials). Rabbit anti-HA (SIGMA; cod. H6908) 1:250 and mouse anti-c-Myc (Santa Cruz Biotechnology; cod. SC40) 1:200 were used as primary antibodies. Images were acquired on Zeiss LSM 510 Meta confocal microscope (Carl Zeiss, Milan, Italy).

#### Statistical methods for the analysis of PLA data

PLA dots were counted on a minimum of tree fields using ImageJ software. Number of dots was normalized for the number of transfected cells in each field. The normalized number of dots was then averaged over the number of field analyzed. Comparison of mean number of dots among GAD67wt/wt, GAD67wt/mut, GAD67mut/mut combinations was performed using ANOVA test followed by *post-hoc* LSD tests.

### Molecular Dynamics

Molecular dynamics simulations were performed starting from the crystal structure available in the Protein Data Bank (https://www.rcsb.org) with code 2OKJ. Details are given in the Supplementary Materials.

### Frequency measure of ultra-rare variants in genes belonging to the GABAergic system

A list of genes involved in GABA synaptic transmission and GABA metabolism was compiled by combining information from REACTOME pathways and Gene Ontology (GO). We searched both repositories using “GABA” keyword and recovered two REACTOME pathways (R-HSA-888590 GABA Synthesis Release Re-uptake Degradation and R-HSA-977443 GABA Receptor Activation) and 7 GO biological pathways/cellular component categories (GO0016917 GABA Receptor Activity; GO0022851 GABA-gated Chloride Ion Channel; GO0032228 Regulation Of Synaptic Transmission GABAergic; GO0050811 GABA Receptor binding; GO0051932 Synaptic Transmission GABAergic; GO0098982 GABAergic Synapse; GO1902710 GABA Receptor). The combined list resulted in 124 non-redundant genes. We limited our study to the 120 genes located on autosomes (Supplementary Table 3).

Among these genes, we measured the frequency of ultra-rare functional mutation in SCZ cases and controls described in dbGaP study phs000473.v1.p2. This cohort included exome sequencing data for 4,969 SCZ cases and 6,245 controls from Sweden population. First, we filtered the dataset as described in^57^. Briefly, we removed variants with GQ < 20 and with more than 10% missing filtered genotypes in either cases or controls. Moreover, we filtered out variants in a homozygous state where observations of the reference allele exceed 5% of the total bases observed at that position, to reduce the number of possible false homozygous calls due to erroneous genotype assignment. This dataset is known to contain a proportion of subjects with substantial Finnish ancestry that can influence rare variants analysis. Thus, we used the method described in^57^ to remove these subjects, resulting in a final dataset of 4,225 cases and 5,834 controls. Filtered variant dataset was annotated using snpEff and GEMINI to obtain the list of missense and LoF (splice, nonsense) substitutions for each gene in each subject. Indels were not considered having a high rate of false positive calls and ambiguous representation of alleles making it hard to compare variants between subjects with public databases. Trying to focus on functional alleles with high impact, we created four variants categories: a) mutations that were ultra-rare (MAF < 0.001) in the considered population; b) mutations that were ultra-rare (MAF < 0.001) in the considered population and rare (MAF < 0.01) in all the human populations from ExAC and 1000 G; c) mutations that were ultra-rare (MAF < 0.001) in the considered population and in all the human populations; d) likely disruptive variants from group c, including only missense variants with CADD phred score > 20, nonsense substitutions and mutations occurring at splice junctions. We measured the number of SCZ cases and control that were homozygous for at least one rare mutation in one of the “GABA system” gene. One-tailed Fisher’s exact test was used to assess if the number of cases harboring at least one rare/ultra-rare mutation in a homozygous state was higher than controls. Then to verify if cases tend to be more homozygous for rare variants in genes of the “GABA system” than in other genes of the genome, we performed a simulation analysis. We generated 5,000 groups of 120 genes by randomly sampling the 18,024 sequenced genes. For each of the 5000 groups, we calculated the difference in the frequency of homozygotes between cases and controls. Simulation p values were calculated as the fraction of the 5,000 simulations obtaining a difference in the frequency of homozygotes equal to or greater than that observed in the original dataset. The simulation was repeated for each variant category.

Moreover, to further exclude that the increased burden of rare mutations in a homozygous state observed in cases could be due to a generalized enrichment of ultra-rare variants in a homozygous state among SCZ cases, we assessed the distribution of ultra-rare mutations in all the genes of the genome excluding the 120 genes of the GABAergic pathway. Then, we used the one-tailed Fisher’s exact test to compare the number of cases and controls that were homozygous for at least one rare variant.

## Electronic supplementary material

Supplementary Materilas
