# Author Correction: A novel homozygous mutation in GAD1 gene described in a schizophrenic patient impairs activity and dimerization of GAD67 enzyme

**DOI:** 10.1038/s41598-020-61779-5

**Published:** 2020-03-10

**Authors:** Chiara Magri, Edoardo Giacopuzzi, Luca La Via, Daniela Bonini, Viola Ravasio, Mohammed E. A. Elhussiny, Flavia Orizio, Fabrizio Gangemi, Paolo Valsecchi, Roberto Bresciani, Alessandro Barbon, Antonio Vita, Massimo Gennarelli

**Affiliations:** 1 Unit of Biology and Genetics, Department of Molecular and Translational Medicine, University of Brescia, Brescia, Italy; 2 Unit of Biotechnology, Department of Molecular and Translational Medicine, University of Brescia, Brescia, Italy; 3 Unit of Physics, Department of Molecular and Translational Medicine, University of Brescia, Brescia, Italy; 4 Neuroscience Section, Department of Clinical and Experimental Sciences, University of Brescia, Brescia, Italy; 5 Department of Mental Health, Spedali Civili Hospital, Brescia, Italy; 6 Genetic Unit, IRCCS Centro S. Giovanni di Dio Fatebenefratelli, Brescia, Italy

Correction to: *Scientific Reports* 10.1038/s41598-018-33924-8, published online 19 October 2018

This Article contains errors in Table 3, where the positions of the coding DNA for the listed mutations are incorrect. The correct Table 3 appears below as Table 1.

**Table 1:** List of rare mutations in genes of the “GABA system” that has been found in a homozygous state among cases or controls.

Genomic	A/U	Gene	Mutation class	Coding DNA	Aa change	Dataset MAF (N. individuals)	Population MAF	CADD
chr2:25044490 T > G	1/0	*ADCY3*	missense	NM_004036.4:c.3023 A > C	E1008A	7.46E-04 (10.055)	2.55E-04	20.5
chr5:170236670 G > A	1/0	*GABRP*	missense	NM_014211.2:c.931 G > A	G311R	2.98E-04 (10.058)	9.01E-05	32
chr8:131880120 C > G	1/0	*ADCY8*	missense	NM_001115.2:c.2182 G > C	A728P	9.94E-05 (10.056)	1.00E-03	18.29
chr11:122929541 T > C	1/0	*HSPA8*	Splice junction	NM_153201.3:c.1324-3 A > G		9.94E-04 (10.055)	3.33E-04	0.66
chr16:76532538 G > A	1/0	*CNTNAP4*	missense	NM_033401.4:c.2312 G > A	R771Q	2.50E-04 (9.986)	7.52E-05	21.7
chr17:39881003 C > T	1/0	*HAP1*	missense	NM_177977.2:c.1810G > A	G604R	1.49E-04 (10.059)	5.22E-02	0.03

