# Supplementary material for: A novel homozygous mutation in GAD1 gene described in a schizophrenic patient impairs activity and dimerization of GAD67 enzyme
Source: Sci Rep. 2018 Oct 19;8:15470. doi: 10.1038/s41598-018-33924-8 (PMC6195539; doi:10.1038/s41598-018-33924-8)
Supplement: Supplementary file 1 — Supplementary Materilas [file 41598_2018_33924_MOESM1_ESM.pdf]

A novel homozygous mutation in GAD1 gene described in a schizophrenic patient impairs activity and dimerization of GAD67 enzyme.

Chiara Magri[1*], Edoardo Giacopuzzi[1], Luca La Via[1], Daniela Bonini[1], Viola Ravasio[1], Mohammed E.A. Elhussiny[1], Flavia Orizio[2], Fabrizio Gangemi[3], Paolo Valsecchi[4,5], Roberto Bresciani[2], Alessandro Barbon[1], Antonio Vita[4,5], Massimo Gennarelli[1,6].

Index

Supplementary Materials..3
References..5
Supplementary Tables..6
Supplementary Figures..15

Supplementary Materials

Mouse primary cortical cultures

Mouse primary cortical cultures were prepared as previously described by [1]. (32). Briefly, mouse cerebral cortices from day 13.5 mouse embryos were mechanically dissociated in cold HBSS containing 10mM HEPES (Invitrogen); the cell suspension was re-suspended in serum-free Neurobasal medium (Invitrogen) supplemented with B-27 (Invitrogen), 30 U/ml penicillin (Sigma-Aldrich, St. Louis, MO, USA), 30 µg/ml streptomycin (Sigma-Aldrich) and 0.5 mM Glutamax (Invitrogen). Neurons were then plated at a density of 30,000 cells/cm^2 on a poly-D-lysine coating (Sigma-Aldrich) in multi-well plates. Three days after plating, 50% of medium was replaced with fresh medium; subsequently, half of medium was replaced once a week for a maximum of four weeks. MOI 5 was used to transduce primary cortical neurons, adding viral particles directly to the culture media.

Animal care was conducted conforming to institutional guidelines that are in compliance with Italian national (DL N116, GU, suppl 40, 18-2-1992) and international laws and policies (European Community Council Directive 86/609, Oja L 358, 1, December 12, 1987; National Institutes of Health Guide for the Care and Use of Laboratory Animals, US National Research Council, 1996).

COS7 cell lines

COS7 cells were cultured at 37°C and 5% CO2 in DMEM medium (Invitrogen) supplemented with 10% of heat-inactivated fetal bovine serum (FBS), 30 U/ml penicillin (Sigma-Aldrich), 30 µg/ml streptomycin (Sigma-Aldrich), 1% minimum Eagle's medium nonessential amino acids, 1 mM sodium pyruvate. MOI 5 was used to transduce COS7 cells, adding viral particles directly to the culture media.

Magnetofection of primary cortical neurons and COS7 cells

Primary cortical cultures (DIV11) and COS7 were transfected using paramagnetic nanobeads (NeuroMag, Oz Biosciences, Marseille, France). The complete medium was replaced before magnetofection with serum-free neurobasal medium. Each plasmid was incubated with NeuroMag beads at a ratio of 1 µg:3.5 µl, in 100 µl of Neurobasal (Life Technologies) for 20 min and added drop-wise to the cultures. The cells were incubated on top of a magnetic plate (Oz Biosciences) for 20 min and the complete medium was restored after 1 h. COS7 were fixed three day after transfection. Neurons were maintained in culture until DIV14 then fixed and used for immunofluorescence experiments.

Proximity Ligation Assay

PLA was performed accordingly to the manufacturer's instructions with minor modifications. Briefly, HEK293T cells previously plated onto glass coverslips were fixed with paraformaldehyde 4% (PFA). Each sample was permeabilized with PBS-Triton 0.3% and then incubated with the blocking solution (Roche™) for about 45 min at room temperature; the primary antibodies incubation was performed overnight at 4°C with rabbit anti-HA (SIGMA; cod. H6908) 1:250 and mouse anti-c-Myc (Santa Cruz Biotechnology; cod. SC40) 1:200. On the following day, samples were washed three times in PBS at room temperature and then cells were incubated 1h at 37°C with PLA probe containing secondary antibodies conjugated with DNA probes. After PLA probe removal, samples were washed 4 times x 10' with Buffer A (Duo-link® PLA Technology® kit) at 37°C. After a brief wash with Buffer A at 37°C, samples were incubated with ligation buffer containing oligonucleotides that hybridize to PLA probe and DNA ligase, which allows annealing between probe and oligonucleotides to form a rolling circle DNA strand. This reaction was incubated for 30 min at 37°C. Subsequently, cells were washed with Buffer A at 37°C and then incubated with amplification-detection solution containing DNA polymerase for rolling circle amplification (100 min at 37°C). Next, samples were washed four times with Buffer B (Duo-link® PLA Technology® kit) at room temperature; then coverslips were incubated for 10 min with mounting buffer containing DAPI and analyzed with a confocal microscope.

Details on molecular dynamics

Molecular dynamics simulations of GAD67wt and mutated isoforms were performed by means of package GROMACS [2,3]. The initial structure for the wild-type dimer was taken from PDB entry 2OKJ. The mutant was built from the wild type, by replacing residue T131 with an alanine. The force field amber99-sb was used, with the PME method for the Coulomb interactions and a Lennard-Jones potential with a cut-off of 10 Å for the short-range interactions. The initial structure was solvated with TIP3P water in a simulation box with a minimum distance of 10 Å between solute and box boundaries. Na+ and Cl− ions were added to reproduce a salt concentration of 150 mM and to neutralize the system. After energy minimization, two equilibration phases followed, at constant volume, and at constant pressure. The subsequent runs were performed at constant temperature (310 K) and pressure (1 atm). For each system, two independent 30-ns simulations were carried out. Secondary structure analysis was performed by means of DSSP (http://swift.cmbi.ru.nl/gv/dssp).

References

1. Lesuisse, C. & Martin, L. J. Long-term culture of mouse cortical neurons as a model for neuronal development, aging, and death. *J. Neurobiol.* **51,** 9–23 (2002).

2. Abraham, M. J. *et al.* GROMACS: High performance molecular simulations through multi-level parallelism from laptops to supercomputers. *SoftwareX* **1–2,** 19–25 (2015).

3. Hess, B., Kutzner, C., van der Spoel, D. & Lindahl, E. GROMACS 4: Algorithms for Highly Efficient, Load-Balanced, and Scalable Molecular Simulation. *J. Chem. Theory Comput.* **4,** 435–447 (2008).

Supplementary Tables

Supplementary Table 1. List of primers

Name	Sequence 5’-3’
GAD1F	CTAAGAACGGTGAGGAGCAAAC
GAD1R	ACCCCATACTTCAAGGTGTCTCT
GAD67_BamHI_F	CGGGATCCCGCCACCATGGCGTCTTCGACCCCATCTTCG
GAD67_SalI_R	GGCGTCGACTTACAGATCCTGGCCCAGTC
GAD67_A391G_F	CAACTATGTCCGCAAGGCATTTGATCGCTCC
GAD67_A391G_R	GGAGCGATCAAATGCCTTGCGGACATAGTTG
GAD67_Myc_STOP_SalI_Rev	ATTGTCGACTTACAGATCCTCTTCTGAGATGAGTTTTTGTTCCAGATCCTGGCCCAGTC
GAD67_HA_STOP_SalI_Rev	ATTGTCGACTTAAGCGTAATCTGGAACATCGTATGGGTACAGATCCTGGCCCAGTC

Supplementary Table 2. List of constructs.

Construct Name	Vectors	Primers used for cDNA amplification	Note	Use
pGAD67-wt	pIRES-hrGFP-1[a] (Agilent)	GAD67_BamHI_F<br>GAD67_SalI_R		Enzymatic activity assay
pGAD67-mut[a]				
PRRLSIN-GAD67wt-HA	pRRLSIN.cPPT.PGK-GFP.WPRE (ADDGENE #12252)	GAD67_BamHI_F<br>GAD67_HA_STOP_SalI_Rev	GFP sequence was removed	Subcellular localization study, PLA assay
PRRLSIN-GAD67mut-HA[a]				
PRRLSIN-GAD67wt-Myc		GAD67_BamHI_F<br>GAD67_Myc_STOP_SalI_Rev		
PRRLSIN-GAD67mut-Myc[a]				

[a] c.391A>G mutation was inserted using primers GAD67_A391G_F and GAD67_A391G_R

Supplementary Table 3. List of genes of the GABAergic system screened for homozygous mutations.

Gene Name	RefSeq	Chromosome
ABAT	NM_000663.4	chr16
ADCY1	NM_001281768.1	chr7
ADCY2	NM_020546.2	chr5
ADCY3	NM_001320613.1	chr2
ADCY4	NM_001198568.1	chr14
ADCY5	NM_001199642.1	chr3
ADCY6	NM_015270.4	chr12
ADCY7	NM_001114.4	chr16
ADCY8	NM_001115.2	chr8
ADCY9	NM_001116.3	chr16
ADORA1	NM_000674.2	chr1
ADORA2A	NM_000675.5	chr22
ADRA1A	NM_000680.3	chr8
ALDH5A1	NM_001080.3	chr6
ARFGEF2	NM_006420.2	chr20
CA2	NM_000067.2	chr8
CA7	NM_001014435.1	chr16

CLSTN3	NM_014718.3	chr12
CNR1	NM_001160226.1	chr6
CNR2	NM_001841.2	chr1
CNTNAP4	NM_001322178.1	chr16
CPLX1	NM_006651.3	chr4
DNAJC5	NM_025219.2	chr20
DNM1	NM_001005336.2	chr9
DRD2	NM_000795.3	chr11
GABARAP	NM_007278.1	chr17
GABARAPL1	NM_031412.2	chr12
GABARAPL2	NM_007285.6	chr16
GABBR1	NM_001319053.1	chr6
GABBR2	NM_005458.7	chr9
GABRA1	NM_000806.5	chr5
GABRA2	NM_000807.2	chr4
GABRA4	NM_000809.3	chr4
GABRA5	NM_000810.3	chr15
GABRA6	NM_000811.2	chr5
GABRB1	NM_000812.3	chr4

GABRB2	NM_000813.2	chr5
GABRB3	NM_000814.5	chr15
GABRD	NM_000815.4	chr1
GABRG1	NM_173536.3	chr4
GABRG2	NM_000816.3	chr5
GABRG2	NM_000816.3	chr5
GABRG3	NM_001270873.1	chr15
GABRG3	NM_001270873.1	chr15
GABRP	NM_001291985.1	chr5
GABRR1	NM_001256703.1	chr6
GABRR2	NM_002043.4	chr6
GABRR3	NM_001105580.2	chr3
GAD1	NM_000817.2	chr2
GAD2	NM_000818.2	chr10
GLRB	NM_000824.4	chr4
GNAI1	NM_001256414.1	chr7
GNAI2	NM_001166425.1	chr3
GNAI3	NM_006496.3	chr1
GNAL	NM_001142339.2	chr18

GNAT3	NM_001102386.2	chr7
GNB1	NM_001282538.1	chr1
GNB2	NM_005273.3	chr7
GNB3	NM_001297571.1	chr12
GNG10	NM_001017998.3	chr9
GNG12	NM_018841.5	chr1
GNG2	NM_001243773.1	chr14
GNG3	NM_012202.4	chr11
GNG4	NM_001098721.1	chr1
GNG5	NM_005274.2	chr1
GNG7	NM_052847.2	chr19
GNG8	NM_033258.1	chr19
GNGT1	NM_021955.3	chr7
GNGT2	NM_001198754.1	chr17
GPR156	NM_001168271.1	chr3
HAP1	NM_001079870.1	chr17
HSPA8	NM_006597.5	chr11
HTR1B	NM_000863.2	chr6
JAKMIP1	NM_001099433.1	chr4

KCNJ10	NM_002241.4	chr1
KCNJ12	NM_021012.4	chr17
KCNJ15	NM_001276438.1	chr21
KCNJ16	NM_001270422.1	chr17
KCNJ2	NM_000891.2	chr17
KCNJ3	NM_001260508.1	chr2
KCNJ4	NM_004981.1	chr22
KCNJ5	NM_000890.3	chr11
KCNJ6	NM_002240.4	chr21
KCNJ9	NM_004983.2	chr1
KIF5B	NM_004521.2	chr10
KRAS	NM_004985.4	chr12
MAF1	NM_032272.4	chr8
NF1	NM_000267.3	chr17
NISCH	NM_001276293.1	chr3
NLGN1	NM_014932.3	chr3
NLGN2	NM_020795.3	chr17
NPAS4	NM_001318804.1	chr11
NPS	NM_001030013.1	chr10

NPY5R	NM_001317091.1	chr4
OXYR	NM_000916,3	chr3
PHF24	NM_001304333.2	chr9
PLCL1	NM_006226.3	chr2
PLCL2	NM_001144382.1	chr3
PPP2CA	NM_002715.2	chr5
PRKCE	NM_005400.2	chr2
PTEN	NM_000314.6	chr10
RAB3A	NM_002866.4	chr19
RAC3	NM_001316307.1	chr17
RIMS1	NM_001168407.1	chr6
SLC32A1	NM_080552.2	chr20
SLC6A1	NM_003042.3	chr3
SLC6A11	NM_001317406.1	chr3
SLC6A12	NM_001122847.2	chr12
SLC6A13	NM_001190997.2	chr12
SNAP25	NM_001322902.1	chr20
STX1A	NM_001165903.1	chr7
STXBP1	NM_001032221.3	chr9

SYN3	NM_001135774.1	chr22
SYT1	NM_001135805.1	chr12
TAC1	NM_003182.2	chr7
TACR1	NM_001058.3	chr2
TRAK1	NM_001042646.2	chr3
TRAK2	NM_015049.2	chr2
USP46	NM_001134223.1	chr4
VAMP2	NM_014232.2	chr17

Supplementary Figures

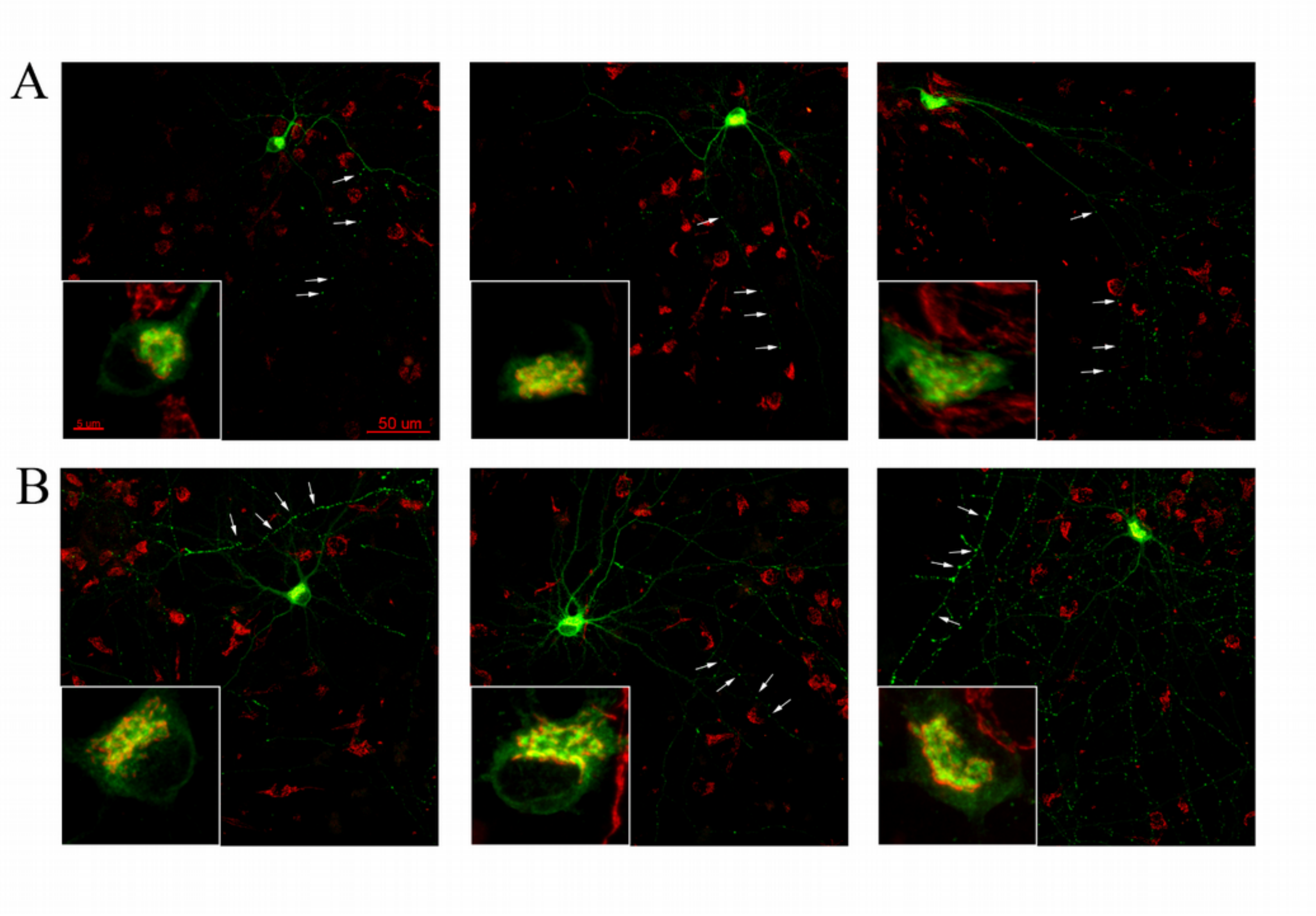

Supplementary Figure 1. Co-immunostaining of GAD67wt or GAD67mut (green signal) and Golgi Network marker GM130 (red signal) in transfected mice primary cortical neurons (scale mar 50 um). In the boxes, magnification of neuronal soma are reported showing the co-localization of GAD76 isoform and Golgi apparatus (scale bar 5 um). White arrows point to axonal clusterization of GAD67 wt and mut form. (A) representative neurons expressing GAD67wt; (B) Representative neurons expressing GAD67mut No difference in intracellular localization has been observed for GAD67wt and GAD67mut forms.

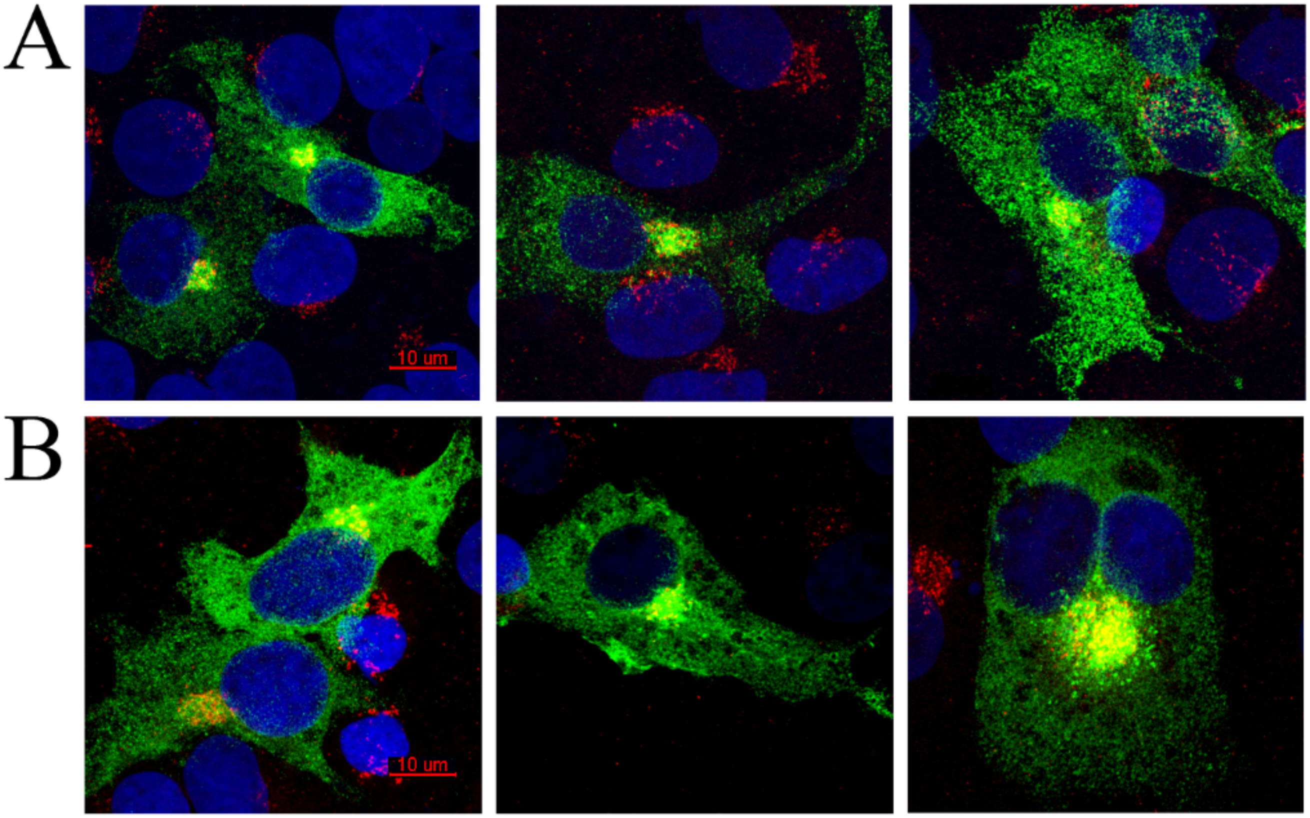

Supplementary Figure 2. Co-immunostaining of GAD67wt (A) or GAD67mut (B) (green) and Golgi Network GM130 marker (red) in transfected COS7 cells. Nuclei were stained using Dapi (blue). Scale bar = 10µm. (A) representative cells expressing GAD67wt; (B) Representative cells expressing GAD67mut. No difference in intracellular localization has been observed for GAD67wt and GAD67mut forms.

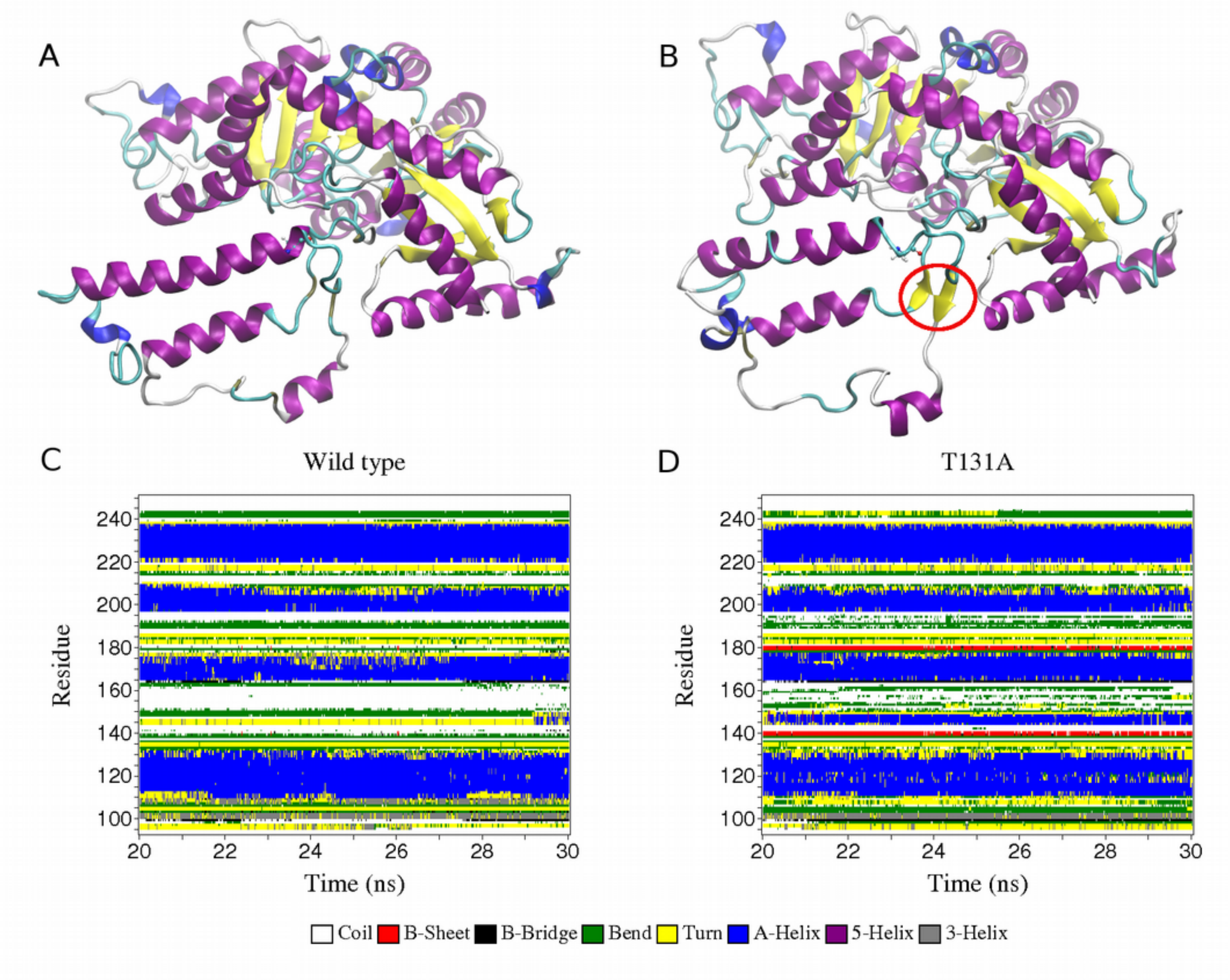

Supplementary Figure 3. Secondary structure of the wild-type and mutant monomer of GAD67 from molecular dynamics. (A) Structure of the wild-type monomer (image generated by VMD, **http://www.ks.uiuc.edu/Research/vmd/**). (B) Structure of the mutant monomer: a red circle shows a portion of β-sheet that is not present in the wild type. (C) Time evolution of the secondary structure of each residue of the wild type. (D) The same as C, for the mutant. The β-sheet component shown in panel B is represented here by the red lines around residues 140 and 180.

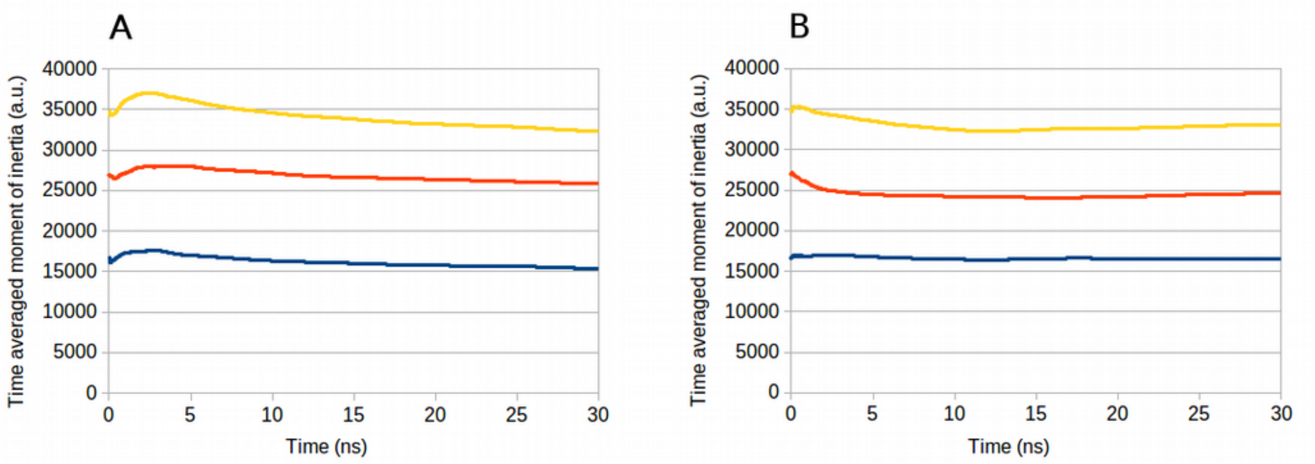

Supplementary Figure 4. Time average of the principal moments of inertia of the region including residues from 93 to 210, as calculated in molecular dynamics simulations of the wild type (panel A) and of the mutant (panel B). The ratios of the three values are different in the mutant with respect to the wild type, showing a change in the shape of this region.

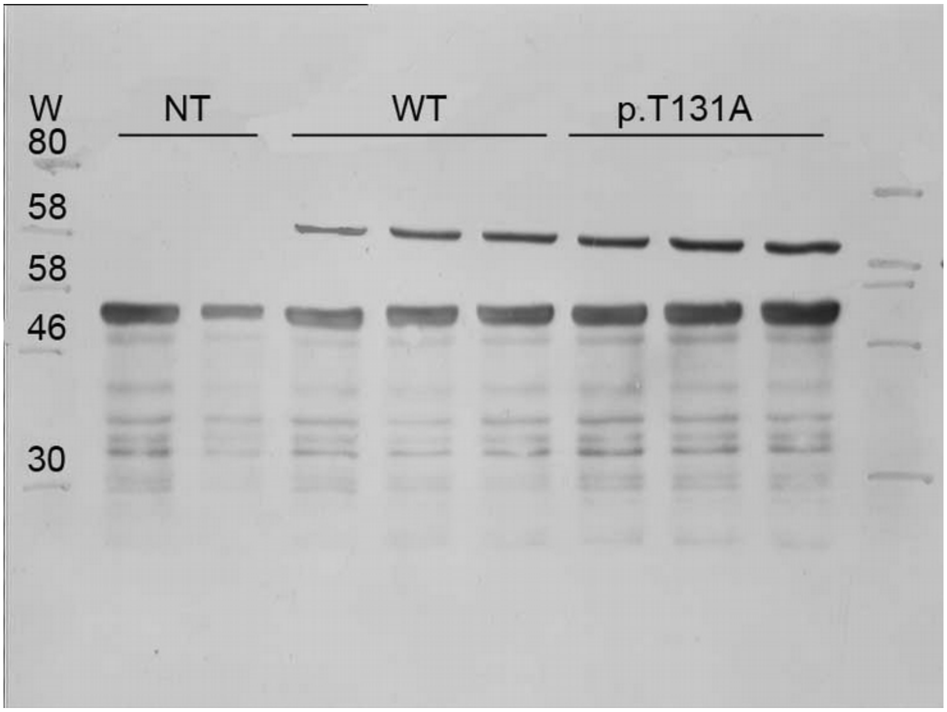

Supplementary Figure 5. Original image of Western-blot staining for GAD67 WT and mutated proteins. Cell extracts from un-trasfected cells (NT), cells transfected with GAD67 WT construct (WT) and mutated construct (p.T131A) were subjected to 12% (w/v) SDS-PAGE and subsequently transferred by electroblotting onto Hybond-P blotting membrane (GE Life Sciences). Membranes were incubated with goat anti-GAD1 or anti-αTub primary antibody and then with donkey anti-goat HRP-conjugated secondary antibody. Detection of immunocomplexes was carried out using SuperSignal West Pico Chemiluminescent Substrate detection kit (Thermo Scientific).
